# Isolated Oculomotor Nerve Palsy Following Minor Head Trauma; a Case report

**Published:** 2017-01-12

**Authors:** Iraj GoliKhatir, Hamed Aminiahidashti, Hasan Motamed Motlagh, Seyed Farshad Heidari

**Affiliations:** Emergency Department, Imam Khomeini Hospital, Mazandaran University of Medical Sciences, Sari, Iran.

**Keywords:** Tomography, x-ray computed, craniocerebral trauma, magnetic resonance imaging, oculomotor nerve diseases

## Abstract

Isolated third nerve palsy develops in numerous intracranial pathologies such as closed head trauma, tumor, and aneurysm. This report describes a 61 years old female with an abrasion on the left forehead and ptosis of the left eye. Initial computed tomography did not reveal any causative cerebral and vascular lesions or orbital and cranial fractures. High-resolution and multi-axial enhanced Magnetic resonance imaging (MRI) can be helpful in diagnosis and monitoring of patients with this rare phenomenon.

## Introduction

Cranial nerve lesions are the result of important kinetic forces to the brain and may develop in the course of rapid acceleration/deceleration, shearing force, skull base injury, and penetrating cranial injury (1). Because of long-term follow-up, repeated surgeries, and reconstructive interventions, cranial nerve injuries have a high rate of morbidity. The trauma that damages the oculomotor nerve is usually severe and associated with other neurologic deficits, basilar skull fracture, orbital injury or subarachnoid hemorrhage (2, 3). However, in rare instances, minor blunt head trauma can cause isolated oculomotor nerve palsy without any other cranial nerve injury. Nevertheless, only a few reports have described the clinical feature of such patients, and mechanisms and imaging studies of nerve damage have not been discussed in depth (4, 5). Here, we report a case of isolated third nerve palsy following minor head trauma.

## Case report:

A 61-year-old woman was transported to the emergency department of Imam Khomeini Hospital, Sari, Iran following a car accident in March 2015. The patient did not experience a decreased level of consciousness and on arrival, she had Glasgow coma scale (GCS) score of 15/15 and did not have amnesia, nausea or vomiting. The patient’s medical history was negative for diabetes, hypertension, hyperlipidemia, smoking, and drug or substance abuse. There was no history of a previous head injury, or any other neurologic or metabolic disorder. On clinical examination, her vital signs were stable. 

**Figure 1:**
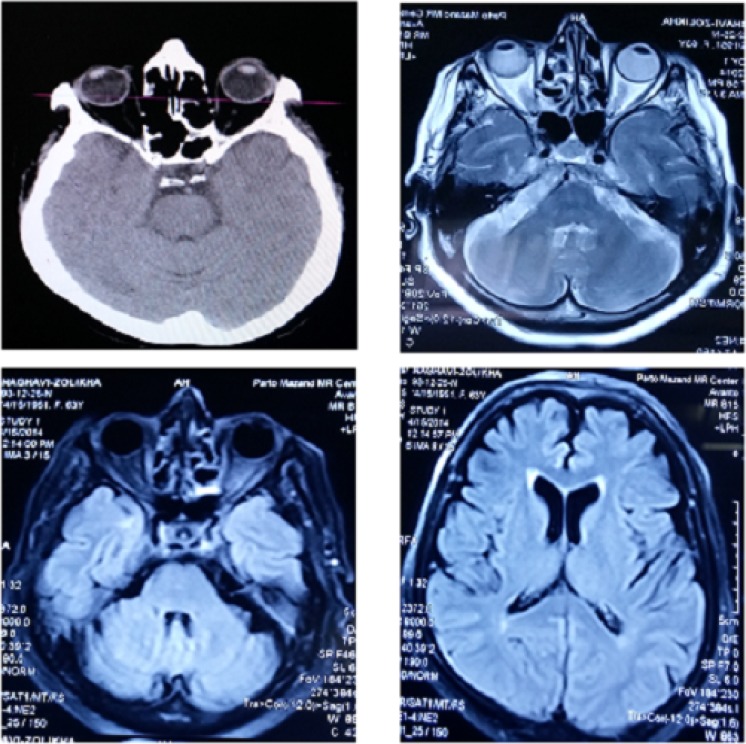
Brain computed tomography scan (top left image) and magnetic resonance imaging of patient.

**Figure 2:**
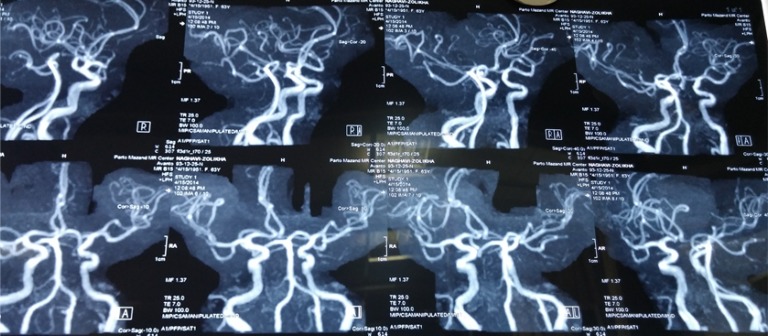
Brain magnetic resonance angiogram of patient.

There was no raccoon eye, battle’s sign, rhinorrhea or otorrhea. She had tenderness, edema, and limitation of left wrist. She had an abrasion on the left forehead and ptosis of the left eye with lateral deviation of the eyeball and a dilated non-reactive pupil that measured 6 mm. The patient could not elevate, depress, or adduct the right eye globe, but the eye intorted on attempted down gaze. Ophthalmologic tests of vision and intraocular pressure were within normal ranges in both eyes. Her left eye had normal range of movement with a normal size reactive pupil. No other abnormality was noted in the neurological exam. Ophthalmological consult ruled out direct trauma to the globe. Brain computed tomography (CT) scan, brain magnetic resonance imaging (MRI) and magnetic resonance angiogram (MRA) were normal (Figures 1 and 2). Four-vessel angiography was normal one week after the head trauma. She was treated with oral prednisone in a tapering dose over several weeks. At the time of follow-up two months after trauma, her ptosis did not improve.

## Discussion

The oculomotor nerve, stems from the frontal surface of mesencephalon, advances forward in the subarachnoid space between the superior and posterior cerebellar artery, and enters the lateral wall of the cavernous sinus by passing through the medial portion of the uncus. At the level of the superior orbital ﬁssure, it divides into the ramus superior and ramus inferior branches. Along this course, fascicules are labeled as the subarachnoid segment, cavernous segment, orbital apex segment, and intra orbital segment, located in the neighborhood of the internal carotid artery, basilar artery and its branches, and the brainstem (-). Head trauma is responsible for 8-16% of oculomotor nerve palsies (-). Tectal hematomas, transtentorial herniation, and isolated oculomotor palsy induced by nerve avulsion or tension at the pontine-mesencephalic junction can develop. Multiple cranial nerve injuries involving the third nerve may occur in skull base fractures involving the cavernous sinus and in maxillofacial or superior orbital ﬁssure injuries; prevalence of isolated third nerve injury due to trauma is reported to be 21% (6, 10, 11). Because traumatic oculomotor nerve palsy is highly associated with skull or cervical spine fracture and intracranial injury, all possible causes and additional injuries should be ruled out with clinical examination and imaging investigation (12). Brain CT and CT angiography are recommended in acutely traumatized patients with oculomotor nerve palsy to rapidly evaluate blood, bone, supratentorial structure, and vascular anomaly. Cerebral MRI is also applied because CT scans may fail to detect abnormalities in the midbrain and the oculomotor nerve itself. On admission, the absence of other neurological signs and normal brain MRI suggested the lesion was most likely within the subarachnoid space, as the other important structures near the oculomotor nerve, such as the brainstem, cavernous sinus and orbit, were intact. In conclusion, the current case had minor head trauma with pure isolated third nerve palsy and a non-reactive dilated pupil, with no abnormal finding on brain CT, MRI, MRA and angiography. As this appears to be a rare case, additional information is necessary to clarify the mechanism in general or specific to this case.
